# The effect of Gonioscopy on keratometry and corneal surface topography

**DOI:** 10.1186/1471-2415-6-26

**Published:** 2006-06-17

**Authors:** Mathew K George, Thomas Kuriakose, Brian M DeBroff, John W Emerson

**Affiliations:** 1 Department of Ophthalmology and Visual Sciences, Yale University, New Haven, CT, USA; 2 Department of Ophthalmology, Christian Medical College, Vellore, TN, India; 3 Department of Statistics, Yale University, New Haven, CT, USA

## Abstract

**Background:**

Biometric procedures such as keratometry performed shortly after contact procedures like gonioscopy and applanation tonometry could affect the validity of the measurement. This study was conducted to understand the short-term effect of gonioscopy on corneal curvature measurements and surface topography based Simulated Keratometry and whether this would alter the power of an intraocular lens implant calculated using post-gonioscopy measurements. We further compared the effect of the 2-mirror (Goldmann) and the 4-mirror (Sussman) Gonioscopes.

**Methods:**

A prospective clinic-based self-controlled comparative study. 198 eyes of 99 patients, above 50 years of age, were studied. Exclusion criteria included documented dry eye, history of ocular surgery or trauma, diabetes mellitus and connective tissue disorders. Auto-Keratometry and corneal topography measurements were obtained at baseline and at three follow-up times – within the first 5 minutes, between the 10^th^-15^th ^minute and between the 20^th^-25^th ^minute after intervention. One eye was randomized for intervention with the 2-mirror gonioscope and the other underwent the 4-mirror after baseline measurements. t-tests were used to examine differences between interventions and between the measurement methods. The sample size was calculated using an estimate of clinically significant lens implant power changes based on the SRK-II formula.

**Results:**

Clinically and statistically significant steepening was observed in the first 5 minutes and in the 10–15 minute interval using topography-based Sim K. These changes were not present with the Auto-Keratometer measurements. Although changes from baseline were noted between 20 and 25 minutes topographically, these were not clinically or statistically significant. There was no significant difference between the two types of gonioscopes. There was greater variability in the changes from baseline using the topography-based Sim K readings.

**Conclusion:**

Reversible steepening of the central corneal surface is produced by the act of gonioscopy as measured by Sim K, whereas no significant differences were present with Auto-K measurements. The type of Gonioscope used does not appear to influence these results. If topographically derived Sim K is used to calculate the power of the intraocular lens implant, we recommend waiting a minimum of 20 minutes before measuring the corneal curvature after gonioscopy with either Goldmann or Sussman contact lenses.

## Background

Accurate biometry is a basic prerequisite for modern-day cataract surgery and is essential to calculate the power of the intraocular lens implant. The axial length, followed by corneal power measurements are the most important factors in intraocular lens power calculations [1]. Both Applanation Tonometry and Gonioscopy are routine office procedures and part of a complete evaluation of the eye. Since contact procedures could affect accurate measurements, we examined whether the act of gonioscopy could change the mean keratometry and corneal topography-based Sim K to such an extent so as to cause a change in the power of the lens implant (based on post-gonioscopy measurements) and, if so, how long such a change would last. Studies have shown that calculations having measurement errors of 0.2 mm in axial length and 0.50 diopter (D) in corneal curvature predict a worst-case primary implant power error of ± 1.17 D [2].

It has been demonstrated that eyelid rubbing causes an immediate and significant change in the Surface Regularity Index (SRI) and the Surface Asymmetry Index (SAI), which return to baseline by the 5th minute after rubbing [3]. It was also shown that Applanation Tonometry did not cause any significant change in mean Keratometry when measured with an Auto-keratometer [4]. A difference in mean corneal refractive power of 0.56 D will result in a 0.50 D discrepancy in the calculated IOL power when the SRK II formula is applied [5]. We chose 0.50 D as a significant difference for the IOL power, because this is the smallest IOL power difference routinely available in our clinical setting. To the best of our knowledge, no study has looked at the effect of gonioscopy on corneal surface topography and keratometry and how long the effect lasts.

## Methods

99 patients (68 male and 31 female) between the ages of 50 and 75 years, presenting to the outpatient department of a tertiary care hospital for cataract surgery, were enrolled in the study. The study was reviewed and passed by the Hospital Ethics Committee, Christian Medical College, Vellore and informed signed consent was obtained from all study participants. Patients with a history of surgical intervention, trauma, dry eye [6], diabetes mellitus and connective tissue disorders were excluded from the study. A drop of hydroxypropyl methylcellulose (Moisol^®^) was instilled in both eyes each time before taking any measurement. Baseline auto-keratometry; Auto-K (KM-500, Nidek, Japan) and Corneal Topography; Sim K (American Megatrends, Tomey, TMS-2, Computed Anatomy, Inc., New York) measurements were obtained from both eyes.

In our study design, one of the patient's eyes was randomized for Goldmann 2-mirror gonioscopy and the other eye was subjected to Sussman 4-mirror gonioscopy. Gonioscopy was always done in the right eye followed by the left eye after instilling a drop of Proparacaine Hydrochloride 0.5% (Paracain^®^). The Goldmann 2-mirror lens (HaagStreit) was inserted as follows [7] in all patients – Methylcellulose was placed on the contact surface of the lens to fill the lens-cornea interface and ensured to be free of air bubbles. After positioning the patient at the slit lamp and dimming room lights, the examiner gently pulled down the lower lid and asked the patient to look up. The inferior lip of the lens was placed beneath the lower lid and the patient was asked to look down. The examiner then raised the upper lid and placed the lens on the eye, while instructing the patient to look directly ahead. The inferior angle was viewed using the superior mirror, followed by the superior angle. The lens was then rotated anticlockwise by 90° to view the nasal and temporal angles, in that order. The gonioscope was then removed from the eye using a slight anticlockwise rotatory motion.

The Sussman 4-mirror lens does not require a coupling fluid. The examiner lifted the upper lid gently and placed the lens over the surface of the cornea. Most of the angle could be viewed without rotating the lens. Approximately 45° of anticlockwise rotation was done to view the areas not covered by the 4 mirrors in the initial position. No attempt was made to indent the corneal surface with the Sussman lens. Both lenses were in contact with the corneal surface for about 45–60 seconds and, in all cases, both the gonioscopies were completed within 60 seconds for each eye. The same person did all 198 gonioscopies using the same instrument for each type of gonioscopy.

Repeat corneal topography and Auto-Keratometry were measured from both eyes within the first 5 minutes, between the 10th – 15th minute and between the 20th – 25th minute after gonioscopy. Each recording was taken after instilling a drop of methylcellulose, to enable uniformity of measurement and to negate the effect of the coupling fluid used with the Goldmann gonioscope. All Keratometry readings were recorded by a single examiner, followed by corneal topography readings by another examiner, using the same instruments. In each case, the right eye was measured prior to the left eye. The readings were taken within 15 seconds of the last blink to negate the effect of prolonged eye opening in anesthetized eyes [8].

The SRK II equation (IOL power = A - 2.5 L - 0.9 K, where A is the A-constant of the lens implant, L is the axial length and K is the mean keratometry) implies that a mean K change of 0.56 Diopters (minimum significant difference for our sample size calculation) results in the IOL power changing by 0.5 Diopters, causing a different IOL to be implanted in the patient. Sample size determination, based on the first 50 patients, used a significance level of 0.05 for a two-tailed test with 80% power to detect a mean K change of 0.56 D. The standard deviation of the pre-gonioscopy Auto-K measurements was 1.37 and the sample size was calculated for an independent-sample T test using SAMPLE (DOS) software (n = 94 per arm). Thus, a total of 198 eyes of 99 patients were included in the study, half of which were randomly subjected to Goldmann 2-mirror gonioscopy and the other half to Sussman 4-mirror gonioscopy. For the paired t-tests using Auto-K measurements, this study has greater power than originally intended. All statistical analyses were done using SPSS 13.0 (Chicago, IL).

## Results

The analysis compared the two groups of eyes that were subjected to the Goldmann and Sussman gonioscopes. The Auto-Keratometer and the Computerized Tomograph were considered to be two separate observers of the changes produced. Baseline Pearson correlation between Auto-K and Sim K measurements were very high for the Goldmann (r = 0.789) and the Sussman (r = 0.817) groups; p = 0.01. Paired t-tests were used to examine the changes in measurements from baseline. Two-sample (independent) t-tests were used to examine differences between the Goldmann and Sussman gonioscopes.

**Figure 1:**
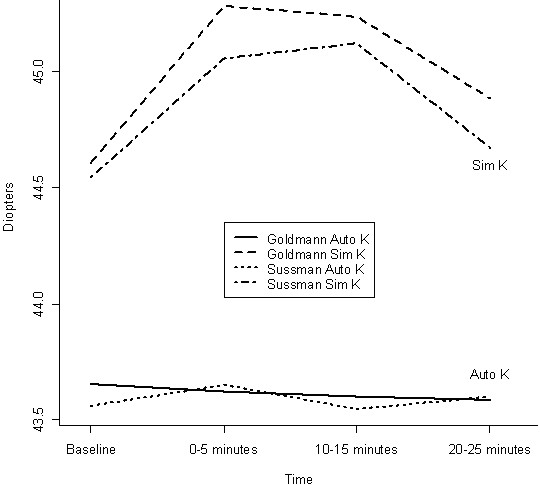
**Change in Mean K after Sussman and Goldmann gonioscopy**. The change in K is measured on the y-axis and the time of measuring changes on the x-axis.

The change in the mean corneal curvature after applying the Goldmann and the Sussman gonioscopes is shown in Table 1 for both Auto-K and Sim K measurements and Figure 1 provides a graphical representation.

**Table 1:** Change in mean K (diopters, D; underscored) from baseline during different time intervals after gonioscopy.

	**Auto K**			**Sim K**		
**Goldmann**	<5 mins	10–15 mins	20–25 mins	<5 mins	10–15 mins	20–25 mins
	
	-0.083 D	-0.025 D	-0.083 D	+0.668 D	+0.633 D	+0.259 D
	CI (-0.176, +0.009)	CI (-0.123, +0.073)	CI (-0.17, +0.004)	CI (+0.164, +1.172)	CI (+0.241, +1.026)	CI (-0.104, +0.622)
	p = 0.077	p = 0.609	p = 0.061	p = 0.010	p = 0.002 *	p = 0.160

**Sussman**	<5 mins	10–15 mins	20–25 mins	<5 mins	10–15 mins	20–25 mins
	
	+0.049 D	-0.018 D	+0.042 D	+0.546 D	+0.557 D	+0.135 D
	CI (-0.022, +0.121)	CI (-0.099, +0.062)	CI (-0.039, +0.123)	CI (+0.045, +1.048)	CI (+0.184, +0.93)	CI (-0.25, +0.52)
	p = 0.173	p = 0.658	p = 0.303	p = 0.033	p = 0.004 *	p = 0.487

CI = 95% confidence interval for the change. p = p-values; mins – minutes; * statistically significant using Bonferroni correction.

The results show a significant steepening in Sim K up to the first 15 minutes using both the Goldmann (p = 0.002) and the Sussman (p = 0.004) gonioscopes. There were no significant differences in the effects produced between the gonioscopes at any time interval after gonioscopy (Sim K at 0–5 min p = 0.638 and Auto K at 0–5 min p = 0.879). Table 2 shows stronger correlations between the two instruments at baseline than at other times. The correlation drops to 0.481 in the first 5 minutes after gonioscopy and increases again at subsequent time intervals. We used the Bonferroni adjustment for multiple comparisons (twelve tests in Table 1) and found significant changes from baseline using Sim K in both the Goldmann and Sussman groups for the 10–15 minute interval after gonioscopy.

**Table 2:** Pearson correlation coefficient (C) between the Auto K and Sim K measurements for both gonioscopes.

	**Time**	C*
**Goldmann**	PreGonio	0.789
	<5 mins	0.481
	5–15 mins	0.619
	20–25 mins	0.611

**Sussman**	PreGonio	0.817
	<5 mins	0.538
	5–15 mins	0.636
	20–25 mins	0.740

* Correlation is significant at the 0.01 level; mins – minutes

## Discussion

With current surgical trends using the latest advances in intraocular lenses and techniques, a patient population with increasingly higher expectations and busy outpatient clinics, the need for accurate and reproducible pre-operative measurements is increasingly important. Gonioscopy is often performed if there is a suspicion of glaucoma, history of uveitis, or a need to evaluate for an anterior chamber IOL. Thus, it becomes important to know how soon one can complete all the required pre-op cataract assessments without compromising on the validity of biometric measurements that might follow. Our study shows that corneal topography-based Sim K, which showed changes after gonioscopy, possibly returns to normal or clinically insignificant levels after 20 minutes. The Auto K was either too insensitive to measure the changes or the changes produced in the area measured were too small to be of any clinical significance. To illustrate the clinical significance, we have given an example (Table 3). This example considers the new power of the IOL calculated using the SRK II formula using the post-gonioscopic altered K readings. The pre-gonioscopic lens power calculated is a 20D lens. One can see that there is a clinically significant (< 0.5 Diopter) change in the IOL power only with Sim K values, up to 15 minutes, resulting in potentially erroneous lens powers if these values were used.

**Table 3:** Possible IOL outcomes after gonioscopy compared to a pre-gonioscopy lens power of +20 Diopter (illustration)

	**Auto-K**			**Sim-K**		
**Goldmann**	<5 mins	5–15 mins	20–25 mins	<5 mins	5–15 mins	20–25 mins
	
	+20.075	+20.023	+20.075	+19.399 ^§^	+19.43 ^§^	+19.767

**Sussman**	<5 mins	5–15 mins	20–25 mins	<5 mins	5–15 mins	20–25 mins
	
	+19.956	+20.016	+19.962	+19.509 ^§^	+19.499 ^§^	+19.879

^§^Clinically significant change when the figures in Table 1 are used for IOL calculation; mins – minutes

The type of keratometric change that occurs after performing gonioscopy is a steepening of the cornea, which is more pronounced with the Goldmann lens, although the difference was not statistically significant. Some difference could be expected because the Goldmann has a more concave contact surface (12 mm/6 mm radius; Haag-Streit, personal communication) and the cornea tends to mould into it. The Sussman has a 9 mm contact surface with a mild degree of convexity. The lack of correlation soon after gonioscopy between the Auto K and corneal topography readings is likely due to the two instruments measuring different parts and data points on the cornea. Conventional keratometry measures the radius of curvature of the central cornea from a diameter of 3 mm using 3 or 4 data points. The asymmetry and asphericity of the cornea are not taken into account. Sim K values are calculated using numerous diopteric data points on 3 mire rings of approximately 3 mm radius and thus are more sensitive to changes induced on the corneal surface.

**Figure 2:**
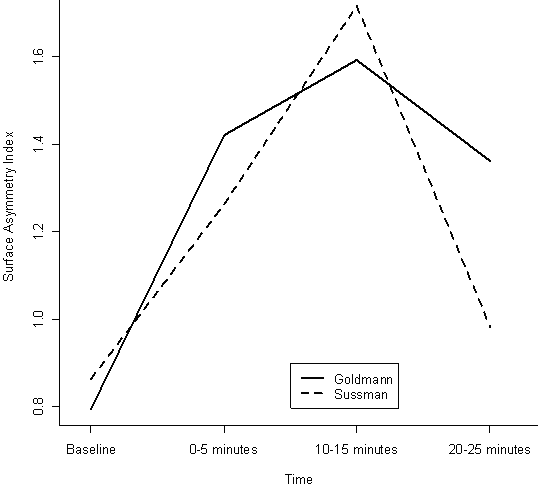
**Change in Mean Corneal Surface Asymmetry Index(SAI) after Sussman and Goldmann gonioscopy**. The change in SAI is measured on the y-axis and the time of measuring changes on the x-axis.

For a single corneal topography reading, imaging is best performed before artificial tear application [9]; however, we decided to instill a tear drop before each measurement to maintain comparability between the two eyes and serially in the same eye. It is possible that the additional tear film induced changes on the corneal surface that were picked up by topography alone. Because we used the tear film in every patient prior to every measurement, it was not possible to calculate the independent effect of the tear film. Surface Regularity (SRI) and Asymmetry (SAI) indices rose significantly after using both lenses. The change in mean SAI is shown in Figure 2.

It has been shown that standard keratometry readings are more accurate and reproducible than topographically derived readings [10]. Based on our findings, the effect of gonioscopy on the mean K derived from Auto-Keratometry is insignificant, but the same cannot be said when relying on topographically derived Sim K. Although arguments have been made both for and against the use of Automated Keratometry in the clinical setting[11,12], we believe that lower variability, robustness to routine clinical measurements [4] and ease of use makes it very suitable for a busy practice.

The effect of reduced blinking rate on the tear film because of the topical anesthetic could account for the highly significant Sim K changes from baseline noted in the 10–15 minute interval [8] although we tried to minimize this by measuring within 15 seconds of the last blink. It was interesting to note that the Surface Asymmetry Index was also highest in this time interval (Fig 2). When interpreting these results, it is important to note that the measurements were taken after instillation of artificial tears, which is not a usual practice in clinics.

## Conclusion

This study examines whether the act of gonioscopy would alter the corneal surface (as measured by Auto K or Sim K) to such an extent that it will cause a change in the power of the IOL calculated for the patient. We also consider the duration of such a change and compare two gonioscopes commonly used in clinical practice.

Corneal Topography-based Sim K showed substantial steepening of the cornea in the first 5 minutes and a significant difference from baseline in the 10–15 minute time interval after both gonioscopes. These changes returned to clinically insignificant levels after 20 minutes. No clinically or statistically significant changes from baseline were recorded with the Auto Keratometer. The effects produced by both Goldmann and Sussman lenses are similar, assuming indentation of the corneal surface is not performed.

When evaluating a patient in the clinic, it would be ideal to record biometric measurements prior to any contact procedure. Short of that, we would recommend waiting for a minimum of 20 minutes before assessing corneal topography in an eye that has just undergone gonioscopy.

## Abbreviations

Auto K: Hand-held Automated Keratometry

Sim K: Computerized Video Tomography-based Simulated Keratometry

IOL: Intraocular Lens implant

## Competing interests

The author(s) declare that they have no competing interests.

## Authors' contributions

MKG was responsible for conceptualizing the study and its design, obtaining patient consent with the help of technicians, writing up the initial manuscript and revising it. TK was responsible for revising the design and critically reviewing the conduct of the study and the manuscript. BMD was responsible for reviewing the manuscript and adding valuable comments to it. JWE was responsible for statistical advice, plots and critically reviewing the manuscript. All authors read and approved the final manuscript.

## Pre-publication history

The pre-publication history for this paper can be accessed here:


